# Optogenetic control of iPS cell‐derived neurons in 2D and 3D culture systems using channelrhodopsin‐2 expression driven by the synapsin‐1 and calcium‐calmodulin kinase II promoters

**DOI:** 10.1002/term.2786

**Published:** 2019-01-30

**Authors:** Si‐Yuen Lee, Julian H. George, David A. Nagel, Hua Ye, Gray Kueberuwa, Leonard W. Seymour

**Affiliations:** ^1^ Department of Oncology, Old Road Campus Research Building University of Oxford Oxford UK; ^2^ Institute of Biomedical Engineering, Old Road Campus Research Building University of Oxford Oxford UK; ^3^ School of Life and Health Sciences University of Aston Birmingham UK; ^4^ Department of Cancer Sciences, Manchester Cancer Research Centre University of Manchester Manchester UK

**Keywords:** 3D culture, alginate hydrogel, calcium‐calmodulin kinase II, channelrhodopsin‐2, induced pluripotent stem cell, neural tissue engineering, optogenetics, synapsin‐1

## Abstract

Development of an optogenetically controllable human neural network model in three‐dimensional (3D) cultures can provide an investigative system that is more physiologically relevant and better able to mimic aspects of human brain function. Light‐sensitive neurons were generated by transducing channelrhodopsin‐2 (ChR2) into human induced pluripotent stem cell (hiPSC) derived neural progenitor cells (Axol) using lentiviruses and cell‐type specific promoters. A mixed population of human iPSC‐derived cortical neurons, astrocytes and progenitor cells were obtained (Axol‐ChR2) upon neural differentiation. Pan‐neuronal promoter synapsin‐1 (SYN1) and excitatory neuron‐specific promoter calcium‐calmodulin kinase II (CaMKII) were used to drive reporter gene expression in order to assess the differentiation status of the targeted cells. Expression of ChR2 and characterisation of subpopulations in differentiated Axol‐ChR2 cells were evaluated using flow cytometry and immunofluorescent staining. These cells were transferred from 2D culture to 3D alginate hydrogel functionalised with arginine‐glycine‐aspartate (RGD) and small molecules (Y‐27632). Improved RGD‐alginate hydrogel was physically characterised and assessed for cell viability to serve as a generic 3D culture system for human pluripotent stem cells (hPSCs) and neuronal cells. Prior to cell encapsulation, neural network activities of Axol‐ChR2 cells and primary neurons were investigated using calcium imaging. Results demonstrate that functional activities were successfully achieved through expression of ChR2‐ by both the CaMKII and SYN1 promoters. The RGD‐alginate hydrogel system supports the growth of differentiated Axol‐ChR2 cells whilst allowing detection of ChR2 expression upon light stimulation. This allows precise and non‐invasive control of human neural networks in 3D.

## 1 INTRODUCTION

The adult brain has very limited regenerative capacity, which is insufficient to prevent the progression of neurodegenerative diseases, such as Alzheimer's and Parkinson's disease or restore neuronal function following damage derived from stroke or traumatic brain injury (Vink & Bullock, 2010). Traditional treatments for neurodegenerative diseases and traumatic brain injury mainly rely on drugs to reduce the rate of degeneration (Kikuchi, Uchikado, Morioka, Murai, & Tanaka, 2012; Van der Schyf, 2011). In order to overcome neurological disorder and deficiency, generation of functional neuronal cells in three‐dimension (3D) combined with optogenetic targeting is a cutting‐edge strategy, not only aimed for treatment through tissue implantation and neuromodulation but also as an in vitro model for drug screening and disease modelling.

In neuroregeneration, human pluripotent stem cells (hPSCs) such as embryonic stem cells (ESCs) and induced pluripotent stem cells (iPSCs) are promising cell sources due to their self‐renewal characteristics and their capability to give rise to neural progenitor stem cells and differentiate to all types of neuronal cells. Homogeneous neuronal populations can be obtained through use of a range of differentiation media and protocols, however, it is often a challenge to generate electrophysiologically active neurons. Many studies have demonstrated efficient differentiation of hPSC and neural progenitor cells to dopaminergic neurons and astrocytes (DA) in vitro via various strategies either on 2D monolayer cultures, co‐cultures with stromal cell lines (PA6 cells), or on 3D extracellular matrices (Cho et al., 2008; Murphy, Laslett, O'Brien, & Cameron, 2017). However, excitatory glutamatergic neurons involved in synaptic functions have not been extensively investigated in tissue engineering and specific‐cell targeting. Specific targeting of glutamatergic neurons in the mixed neuronal population is made possible by using an optogenetic approach.

Optogenetics is a novel technology that integrates genetic and optical engineering to achieve both high temporal and spatial precision within neuronal tissues, overcoming many of the limitations of conventional electrical stimulation and pharmacological methods. Optogenetics facilitates targeting and manipulation of specific neuronal sub‐populations, such as excitatory and inhibitory neurons. Moreover, the brain tissues of conscious animals can be controlled by the expression of light‐sensitive genes such as channelrhodopsin‐2 (ChR2), which triggers membrane depolarisation upon illumination with 470 nm wavelength blue light. ChR2 has been applied to neuroscience research for imaging, targeting specific neurons within neural networks, monitoring neural developments, and regulating neural network activity (Steinbeck et al., 2015). In stem cell engineering, optogenetics has been used for tracking the differentiation of stem cells, for functional analysis of embryonic stem cell‐derived grafts, as well as for testing the functional integration of induced pluripotent stem cell‐derived neurons (Colasante et al., 2015).

Efficient gene delivery and expression of ChR2 in neuronal cells has been demonstrated using lentiviral vectors with neuron‐specific promoters (Hioki et al., 2007). Lentiviral vectors with five different types of neuron‐specific promoter: synapsin I (SYN1), calcium/calmodulin dependent protein kinase II (CaMKII), tubulin alpha I (Ta1), neuron‐specific enolase (NSE), and platelet‐derived growth factor‐beta chain (PDGF) have been generated by fusing neuron specific elements with the cytomegalovirus enhancer element (Gloster et al., 1994; Lee et al., 2010; Sasahara et al., 1991). Among these promoters, CaMKII is specifically active in glutamatergic neurons, whereas pan‐neuronal promoters are active in all neural types. In this study, CaMKII and SYN1 promoters were selected to establish optogenetic control of hPSC‐derived neural networks. This led to the downstream induction of ChR2 expression facilitating the subsequent effect on the regulation of neural activity of hPSC‐derived neural networks to be compared.

Furthermore, generation of neurons in 3D requires bioactive scaffolds combined with support cells to maintain hPSC viability and neural differentiation, leading to a functional neural network that imitates those in the human brain. It is a challenge to improve the microenvironment of 3D culture for optimal survival of optogenetically engineered cells. Hydrogels have been reported as efficient cell carriers or growth factor delivery vehicles in brain tissue engineering (Cheng, Chen, Chang, Huang, & Wang, 2013). In addition, their mechanical properties are comparable with those of human brain tissue (Aurand, Wagner, Lanning, & Bjugstad, 2012), being soft and supportive for cell growth. Cell encapsulation in a hydrogel is, therefore, a promising therapeutic approach that can be applied for culture in vitro or transplanted in vivo.

In this study, alginate hydrogel was utilised for 3D culture of optogenetically modified neurons derived from hPSCs. Previous studies have demonstrated that human embryonic stem cells (hESCs) can be propagated and differentiated in 3D culture using alginate microcapsules (Chayosumrit, Tuch, & Sidhu, 2010; Dean, Yulyana, Williams, Sidhu, & Tuch, 2006). Alginate supports the survival of encapsulated cells in high‐density cultures through allowing rapid exchange of nutrients, oxygen, and stimuli across the outer layer of the micro‐capsule. Encapsulated cells are buffered in vivo from external factors such as antibodies from the host (Serra et al., 2011). Furthermore, it has been shown that the alginate encapsulation promotes cell growth, differentiation, maturation, and protein secretion of various cell types including mesenchymal stem cells, mouse ESCs, and human ESCs (Addae et al., 2012; Serra et al., 2011). Despite success in the growth support of many cell types, studies utilising alginate encapsulation for hiPSC differentiation and 3D culture are limited (Addae et al., 2012; Chayosumrit et al., 2010).

In order to improve the ability of alginate hydrogel to support cell attachment and differentiation, alginate hydrogel has been functionalised with Arginylglycylaspartic acid (RGD) peptide. The RGD peptide sequence is found within many cell adhesive proteins within the extracellular matrix, acting as an integrin binding sites that mediate cell adhesion and increase cell‐matrix interaction (Ruoslahti, 1996). The physical conformation of the RGD loop regulates the affinity of integrin binding, having a direct effect on cell adhesion (Hersel, Dahmen, & Kessler, 2003). RGD and other peptide ligands such as YIGSR (Tyr‐Ile‐Gly‐Ser‐Arg) and IKVAV (Ile‐Lys‐Val‐Ala‐Val) have been reported as crucial in neural progenitor cell survival and differentiation (Li et al., 2014; Villard et al., 2006).

Improving cell integrin within the alginate hydrogel alone is insufficient for optimal culture of hPSCs. Cell survival within alginate hydrogel, particularly hPSCs, can be further enhanced by inhibiting apoptosis using individual or multiple small molecules such as through the use of Rho‐associated kinase inhibitor (ROCKi, Y‐27632). The use of this small molecule was found to increase the viability of dissociated hESCs (10 μM) and several cell lines such as glioblastoma (U87‐MG), patient‐derived glioblastoma xenoline (JX12), primary glioblastoma (SMC448), and non‐cancerous astrocytes (Lau, O'Shea, Broberg, Bischof, & Beart, 2011; Tilson et al., 2015; Watanabe et al., 2007). The anti‐apoptotic effect of Y‐27632 in feeder‐free culture, and its effect in pro‐expansion of most stem cell types has been successfully demonstrated (Watanabe et al., 2007), however, the incorporation of ROCKi into the alginate hydrogel had not been previously applied.

The aim of this study was to investigate the use of neural sub‐population specific promoters to drive ChR2 expression in hiPSC‐derived neurons (Axol‐ChR2) after being encapsulated in the functionalised alginate hydrogel (RGD + ROCKi). We examined whether these cells survived and matured into active neurons/neural networks that respond to light stimulation, both in 2D and 3D culture. Results demonstrate that cells survive with optical excitability and show neural calcium‐flux functionality in both 2D and 3D culture systems.

## 2 MATERIALS AND METHODS

### 2.1 Culture and differentiation of human pluripotent stem cells

Human embryonic stem cells, (HUES2 cell line) were obtained from Harvard University, Melton Lab, Massachusetts, United States. The use of HUES2 to conduct human stem cell research was approved by the ethical committee at the University of Oxford. The cells (1 × 10^6^ cells/ml in hESCs medium) were co‐cultured on a mouse embryonic fibroblast feeder layer and incubated at 37°C without disturbance. Cells were treated with ROCKi, Y‐27632 (Calbiochem, California, United States) at 10 μM. Medium was changed daily (replaced with 50% of fresh medium) for 6 days before subculture or transfer to matrigel (feeder free culture) in mTeSR1 medium (Stemcell Technologies Inc., France). The mTeSR1 medium was changed daily until hESC cells reached 80% confluence, cells were then passaged using accutase to create single cell suspensions (Invitrogen, United States). Cells were expanded in culture prior to encapsulation with alginate hydrogel. In contrast, human induced pluripotent stem cell‐derived neural progenitor cells (hiPSC‐NPC; AXOL13 and AXOL15 cell lines), purchased from Axol Bioscience, Cambridge, United Kingdom. HiPSC‐NPC cells (50,000 cells/cm^2^) were plated onto laminin coated six‐well plates (Sigma, United States) in Axol plating‐XF medium for 24 hr. Cultures were maintained in Axol neural maintenance‐XF medium (up to 50 days) until differentiation in Axol neural differentiation‐XF medium for 72 hr to obtain mature neurons.

### 2.2 Preparation of primary neurons

The dissociated hippocampal cells were gifts from Fabio Biachi (Oxford University), isolated from 14.5‐day pregnant mice according to the local established ethical policies. The individual embryos were separated by cutting uterus between embryo locations and kept in cold neural basal media (NB media). An incision was made in embryo head to dissect the brain in half via the sagittal midline. The meninges were removed, and the hippocampus was removed into NB media on ice. Hippocampi in NB media were centrifuged at low speed (500 rcf) for 2 min following by enzymatic digestion with trypsin for 25 min at 37°C and stirred every 5 min to create a homogeneous mixture. Enzymatic activity was then neutralised, and the tissues were triturated using a fine Pasteur pipette coated with fetal bovine serum to obtain a single cell suspension. Cells were centrifuged and resuspended in complete NB media prior plated on PLO/laminin coated dishes for expansion.

### 2.3 Cell modification using optogenetics

The channelrhodopsin‐2 (ChR2) gene was fused to enhanced yellow fluorescent protein (eYFP) and cloned into a lentivirus expression plasmid with cell type‐specific promoter (a) pan‐neuronal promoter, pLenti‐synapsin1‐hChR2‐(E123T‐T159C)‐EYFP‐WPRE and (b) glutametergic neuron promoter, pLenti‐CaMKII‐hChR2‐(E123T‐T159C)‐EYFP‐WPRE, driving ChR2‐eYFP expression in the specific cells. The universal or constitutive promoter of human origin, human elongation factor‐1 alpha (EF1a), was included in the study. The transfer vectors of ChR2 constructs were a gift from Dr David Nagel. Lentiviral production was performed according to the standard protocol. Viral particles were centrifuged at 500 rcf for 10 min, then resuspended in OPTI‐MEM, filtered, and titrated using an enzyme immunoassay, HIV p24 antigen ELISA (Cell Biolabs' QuickTiter™ Lentivirus Titer Kit). The hiPSC‐NPCs (AXOL13) were incubated with respective amount of viruses (multiple of infection‐1 [MOI‐1] and multiple of infection‐2 [MOI‐2]) for first infection in 24 hr and second infection in the following 24 hr. Transduction efficiency of differentiated ChR2‐positive Axol cells (Axol‐ChR2) was evaluated using flow cytometry and fluorescent microscopy after 14 days of transduction.

### 2.4 Flow cytometry

Axol‐ChR2 cells: Axol cells transduced with lentiviral vectors encoding the ChR2 gene under control of the SYN1, CaMKII, and EF1a promoter, were produced by transduction at MOI‐1 and MOI‐2. Cells were enzymatically detached from culture at Days 7, 14, and 28, washed with culture medium, and immersed in phosphate buffer saline (PBS, pH = 7.4) before quantifying the level of transduction by flow cytometry (BD FACSCalibur). Untreated cells or non‐transduced cells were used as a negative control to establish positive gate regions. Positive cells were those in positive gate regions, the proportion of positive cells as a percentage of live cells is given. A minimum total of 10,000 live events were acquired for each sample. Data were analysed with BD CellQuest™ Pro Software.

### 2.5 Immunofluorescent staining

Optogenetically engineered hiPSC‐NPCs were differentiated to neurons (Axol‐ChR2 cells containing SYN1/CAMKII promoter) and fixed with 4% paraformaldehyde in PBS for 30 min at room temperature (RT). The cells were permeabilised and blocked (5% BSA, 0.2% Triton‐X100, and 0.1% tween 20 in PBS) for 1 hr and incubated overnight in primary antibody solution at 4°C. Cells were washed three times with wash buffer (0.2% BSA, 0.2% Triton‐X100, and 0.1% Tween 20 in PBS) and then blocked with 10% goat serum in wash buffer for 30 min. Secondary antibody solution was added, and cells were incubated at RT for 2 hr. Finally, the cells were washed with washing buffer and stained with 300 μl of 6‐diamidino‐2‐phenylindole (DAPI). Pictures were taken using confocal microscopy (Zeiss‐LSM 780, Germany) and analysed with ZEN light 2013 software.

The primary antibodies used were mouse anti‐neuron‐specific class III beta‐tubulin (Tuj1 for neural cytoskeleton; 1:1,000; abcam, Cambridge, United Kingdom, www.abcam.com), mouse anti‐γ‐aminobutyric acid B receptor 1(GABA‐B‐R1 for inhibitory neurons; 1:50; abcam), rabbit anti‐S100B (1:200; abcam), rabbit anti‐glial fibrillary acidic protein (GFAP for astrocytes; 1:2500; abcam), and rabbit anti‐vesicular glutamate transporter 1 (vGlut1 for excitatory neurons; 1:100; abcam). Secondary antibodies: Alexa Fluor®568‐conjugated goat anti‐mouse, Alexa Fluor®649‐conjugated goat‐anti rabbit, and Alexa Fluor®488‐conjugated goat anti‐rabbit were all used at 1:500 (Invitrogen, Life Technologies, Grand Island, New York, www.lifetechnoogies.com). Negative controls for the samples were prepared in the same staining protocol, containing no primary antibody but only secondary antibody.

### 2.6 Fabrication and functionalisation of alginate hydrogel

High molecular weight ultrapure alginate (Pronova‐MVG) was obtained from Pronova Biomedical, Oslo, Norway (Mw = 231 kDa, high guluronic acid content, medium viscosity >322 cP). Alginate was dissolved in 0.9% (w/v) sodium chloride (NaCl) at 60°C for 6 hr, and autoclaved. A stock solution of 2.0% (w/v) was prepared and diluted to 1.2%, 1.4%, 1.6%, and 1.8% (w/v), respectively. Alginate solutions were transferred to a 5 ml syringe connected to a syringe pump (Harvard apparatus, United States) at a distance of 7 cm from the gelling bath. To polymerise the gel into beads, the solutions were extruded into 102 mM calcium chloride (CaCl_2_) on a stir plate using 30‐gauge needle at different flow rates (2, 2.5, and 3 ml/min) and gently stirred for 7 min at room temperature. The beads were washed with sodium chloride twice and immersed in PBS for microscopic analysis.

RGD peptide was conjugated to alginate via an amide bond between the terminal amine (NH_2_) of peptide and the carboxylate (COOH) on alginate. The functionalisation of alginate utilised aqueous 1‐ethyl‐3‐[3‐dimethylaminopropyl] carbodiimide (EDC) chemistry (Rowley, Madlambayan, & Mooney, 1999). Sulfo‐NHS (0.1 mmol), EDC (0.2 mmol) and RGD peptide were added into the alginate‐MES buffer, and the reaction was performed for 20 hr. The RGD‐alginate solution was purified by dialysis (3,500 molecular weight cut‐off) against distilled water (dH_2_O) in decreasing salt solution concentrations for 72 hr, then lyophilised for 24 hr and stored desiccated until use. The RGD‐alginate was incorporated with ROCKi (10 μM) in the culture medium during cell encapsulation.

### 2.7 Characterisation of alginate hydrogel

#### 2.7.1 Physical measurement

Alginate beads were characterised using microscopic analysis (Nikon eclipse Ti, Japan) to measure the bead size. Mean diameter (*n* = 50) was calculated to determine the bead size at different concentrations (1.2%, 1.4%, 1.6%, 1.8%, and 2.0%) and flow rates (2, 2.5, and 3 ml/min).

#### 2.7.2 Cell viability assay

The hESCs (HUES2 derived from feeder‐free layers) and hiPSC‐NPC cells (Axol) were harvested as single cells using accutase, resuspended in 1.8% alginate and RGD‐alginate solutions, prior to extrusion into 102 mM CaCl_2_ at a flow rate of 3 ml/min. Beads encapsulated with hESCs or Axol cells were rinsed with PBS twice, transferred to a six‐well dish and cultured in a chemically‐defined condition (mTeSR1 medium containing ROCKi was used for hESCs whereas Axol neural maintenance‐XF medium containing ROCKi was used for Axol cell culture). The constructs were removed from culture medium (500 μl per well), stained with calcien‐AM and propidium iodide (PI) solution (Sigma Aldrich, Missouri, United States) at 37°C for 1 hr. The constructs were rinsed and immersed in PBS for confocal imaging (Zeiss‐LSM 710) using ZEN light 2011 software. Pictures were taken at the equatorial plane using a 5x objective. The percentage of surviving cells was determined by dividing the number of cells stained green by the total number of cells (stained green and red) at a sectional‐view using ImageJ software (National Institutes of Health, Bethesda, Maryland). The experiments were carried out in triplicate, and results were analysed using Prism5.

### 2.8 ChR2 stimulation and calcium imaging

Live calcium imaging was performed with CAL‐590 (AM, Mw‐1129.86, ATT Bioquest). Transduced and nontransduced Axol cells and primary neurons (positive control) were incubated with CAL‐590 AM at a concentration of 10 μM (solubilised in DMSO and prepared with Pluronic F‐127) in artificial cerebrospinal fluid (ACSF) for 40 min at RT. The cells were washed and incubated in ACSF and imaging was carried out using a Carl Zeiss 780 confocal microscope. The set‐up was as follows: 20x objective, Zoom 1, 512 × 512 format, 8‐bit resolution, xyt scan mode, 400 Hz speed, 1.0 airy pinhole, frame average 1, line average 4 for image, and 1 for time lapse imaging. Optogenetically modified and unmodified cells in 2D and 3D were excited with 540/565 nm laser for ion calcium indicator (CAL‐590 AM), and 488 nm laser (at 20% laser power) to detect the yellow fluorescence from eYFP whereas 100% 488 nm laser power was set to stimulate the ChR2‐eYFP. Time lapse imaging was set with a stimulation of 1.635 s, and a total of 200 frames were recorded at the basal ion calcium level.

### 2.9 Statistically analyses

The data in this study were analysed using SPSS 10 software (SPSS Inc., Chicago, United States). Student's *t* test was used for comparisons between two groups. ANOVA followed by Tukey's post hoc test was used for comparisons among the groups. Statistical significance was accepted at *p* < 0.05, unless otherwise stated. All experiments were performed in triplicate.

## 3 RESULTS

### 3.1 Lentiviruses‐mediated expression of ChR2‐eYFP in neurons derived from human iPSCs (Axol cells)

**Figure 1:**
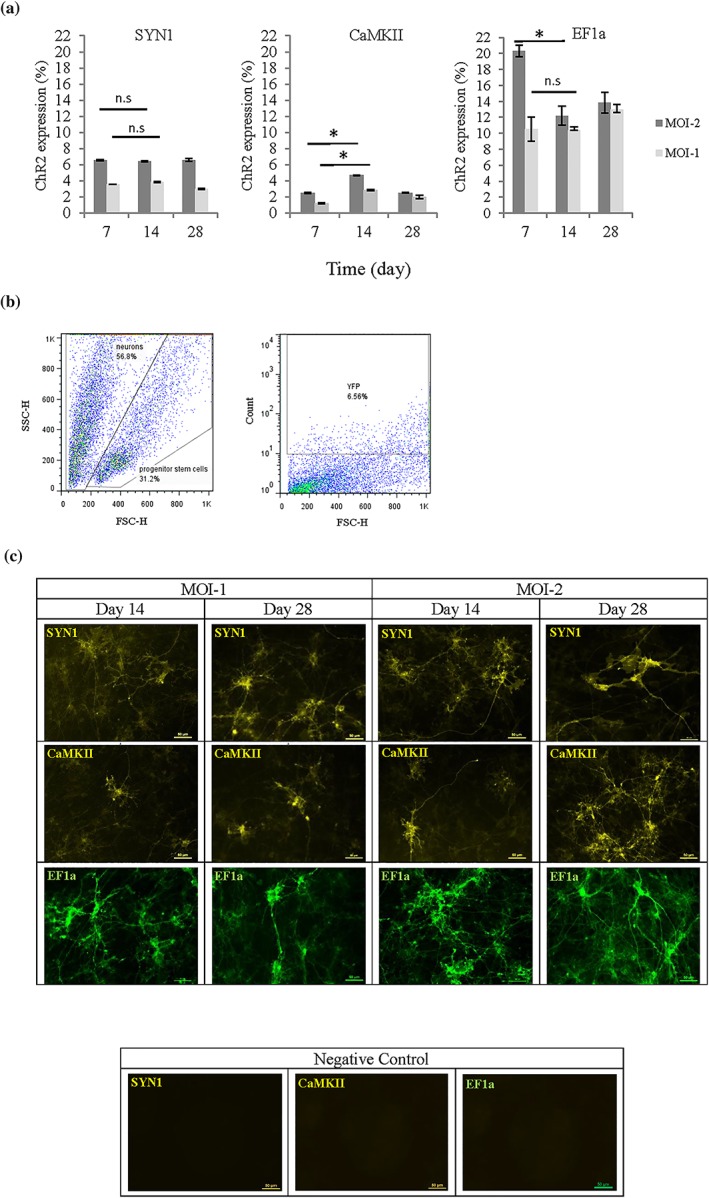
Expression of fluorescent tagged ChR2 in 2D neural cultures of transduced Axol cells under different promoters: channelrhodopsin‐2‐enhanced yellow fluorescent protein‐synapsin‐1 (**SYN1**), ChR2‐eYFP‐calciumcalmodulin kinase II (**CaMKII**), and ChR2‐GFP‐elongation factor‐1 alpha (**EF1a**). (a) Transduction efficiency was assessed using flow cytometry and the graphs show positive ChR2 expression quantified for each time point. Expression of the universal promoter, EF1a was used as a positive control. Axol cells were transduced using different conditions, and with one or two multiplicities of infection (MOI‐1 and MOI‐2). Significance was assessed using ANOVA; * = *p* < 0.05; Error bars denote standard deviation (±*SD*), *n* = 3. (b) An example showing how gating was used to quantify flow cytometry results for eYFP‐ChR2 expression under the SYN1 promoter. Nontransduced cells were used in setting the gating region, and ChR2‐eYFP positive neurons were quantitated. (c) Fluorescent imaging reveals that ChR2 was distributed evenly. Cells were imaged using a fluorescence microscope (Nikon Eclipse Ti‐E, Japan). Negative controls: images of non‐transduced cells taken using identical setting, indicate that positive signal does not arise from non‐specific fluorescence. Scale bar: 50 μm [Colour figure can be viewed at wileyonlinelibrary.com]

The expression level of ChR2‐eYFP was higher in Axol cells driven by the SYN1 promoter (6.5%) than the CaMKII promoter (2–5%) at MOI‐2 from Days 7–28 after transduction (Figure 1a), indicating that the pan‐neuronal promoter SYN1 was a stronger promoter in these cells than CaMKII. The expression resulting from regulation by the positive control universal promoter EF1a led to a two fold (>13%) increase in ChR2‐eYFP expression in comparison with the levels of expression regulated by the SYN1 and CaMKII promoters in Axol cells (Figure 1b). The transduction condition MOI‐2 is found to be more efficient than MOI‐1. On Day 14 of transduction, strong ChR2 signals were expressed (Figure 1a–c), and mature neuronal morphology was indicated by the presence of glutamatergic and GABAergic cells in the differentiated optogenetically‐modified Axol cells. Confocal microscopy showed the distribution of ChR2‐eYFP/GFP expression in the cell membrane and along the dendrites. Results suggested that the neurons needed to be cultured for at least 14 days following transduction before optical stimulation and downstream analyses (Supplementary Figure 1). The differentiated neurons at Day 7 post‐infection lacked maturity, with low neural network activity that made them unsuitable for light stimulation and recording. Factors such as neural maturity, stability of ChR2 expression, and network connectivity needed to be taken into consideration, which required the treated cell to mature in the culture for longer periods.

### 3.2 Optogenetic engineered Axol cells exhibited characteristics of mature neurons

**Figure 2:**
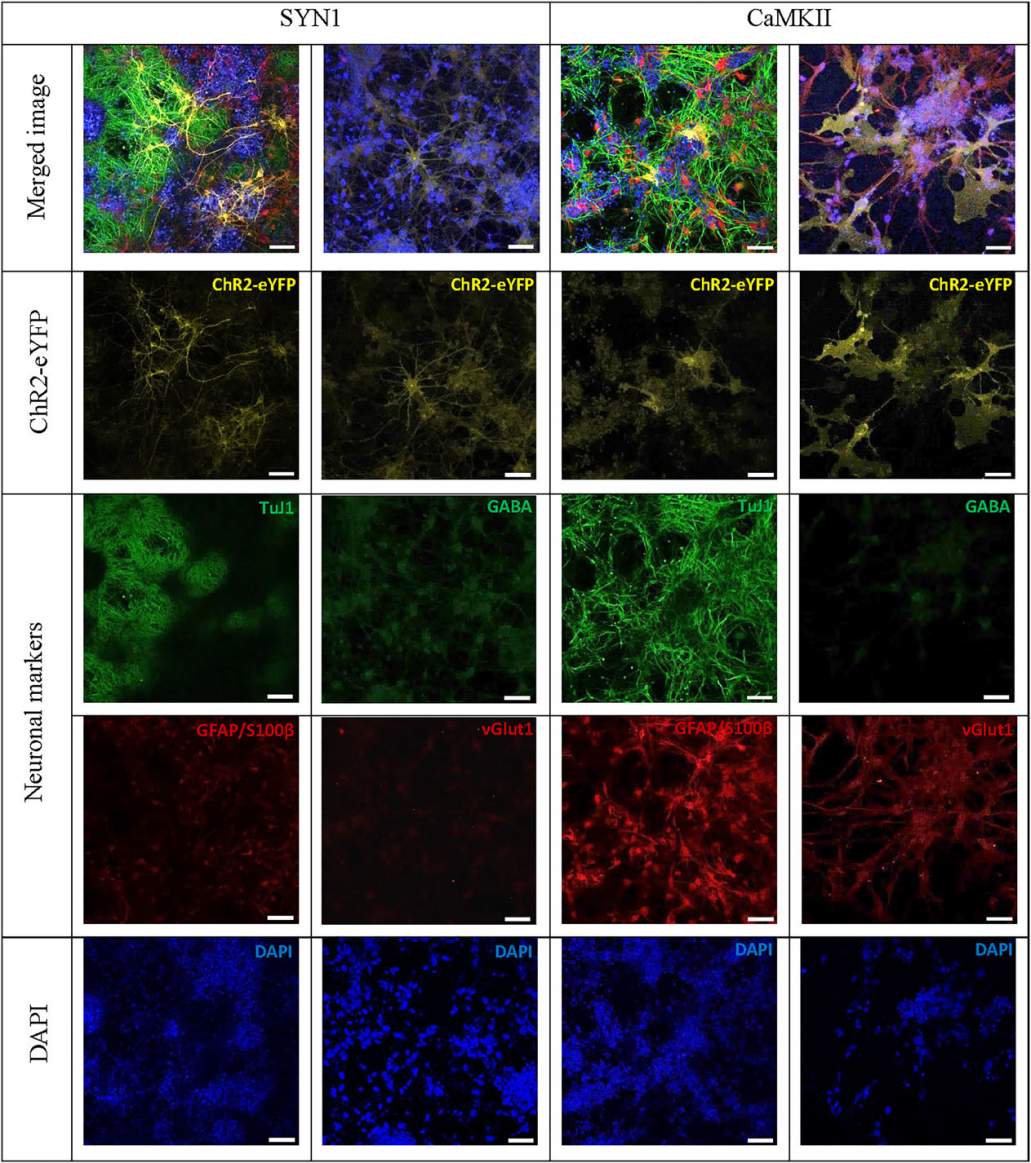
Immunofluorescent staining of human induced pluripotent stem cell‐derived neurons (Axol) demonstrating positive expression of neural markers TuJ1, GFAP/S100β, GABA (low expression under calcium calmodulin kinase II [CaMKII] promoter) and vGlut1 (low expression under synapsin‐1 [SYN1] promoter) under the control of different neuronal‐specific promoters. Axol cells containing pSYN1‐ChR2‐eYFP and pCaMKII‐ChR2‐eYFP were cultured on a laminin coated 24‐well, glass‐bottom plates, and matured in Axol differentiation and maintenance medium for 3–6 weeks. The cells were fixed, permeabilised, and stained with identifying markers shown in green depicting the neural cytoskeletal (TuJ1), astrocytes (GFAP/S100ß), GABAergic neurons (GABA), and glutamatergic neurons (vGlut1). ChR2‐eYFP is depicted in yellow whereas cell nuclei are depicted in blue (DAPI). Fluorescent images were captured using confocal microscopy (Zeiss‐LSM 710) and processed with ZEN light 2011 software. Scale bar: 50 μm [Colour figure can be viewed at wileyonlinelibrary.com]

In order to better characterise cellular phenotype, immunofluorescent staining of Axol‐ChR2 cells containing different promoters (between passages 8 and 10) was performed. The transgene ChR2‐eYFP was expressed in both pSYN1‐ChR2‐eYFP and pCaMKII‐ChR2‐eYFP driven Axol cells. The cells were stained strongly positive for the mature neural markers ßIII‐tubulin (TuJ1) and glial fibrillary acidic protein (GFAP/S100β; Figure 2). High expression of GABAergic cells (GABA) and low signals of glutamatergic cells (vGlut1) were observed in Axol‐ChR2 cells driven by SYN1. Vice versa, high expression of glutamatergic cells (vGlut1) and low signals of GABAergic cells (GABA) were shown in Axol‐ChR2 cells driven by CaMKII (Figure 2). Results indicated successful differentiation of inhibitory and excitatory neurons from hiPSC‐NPCs, with both SYN1 and CaMKII promoters driving the expression of ChR2 in their specific neuronal subtype.

### 3.3 Concentrations and flow rates regulate the diameter of alginate bead in 3D cell culture

**Figure 3:**
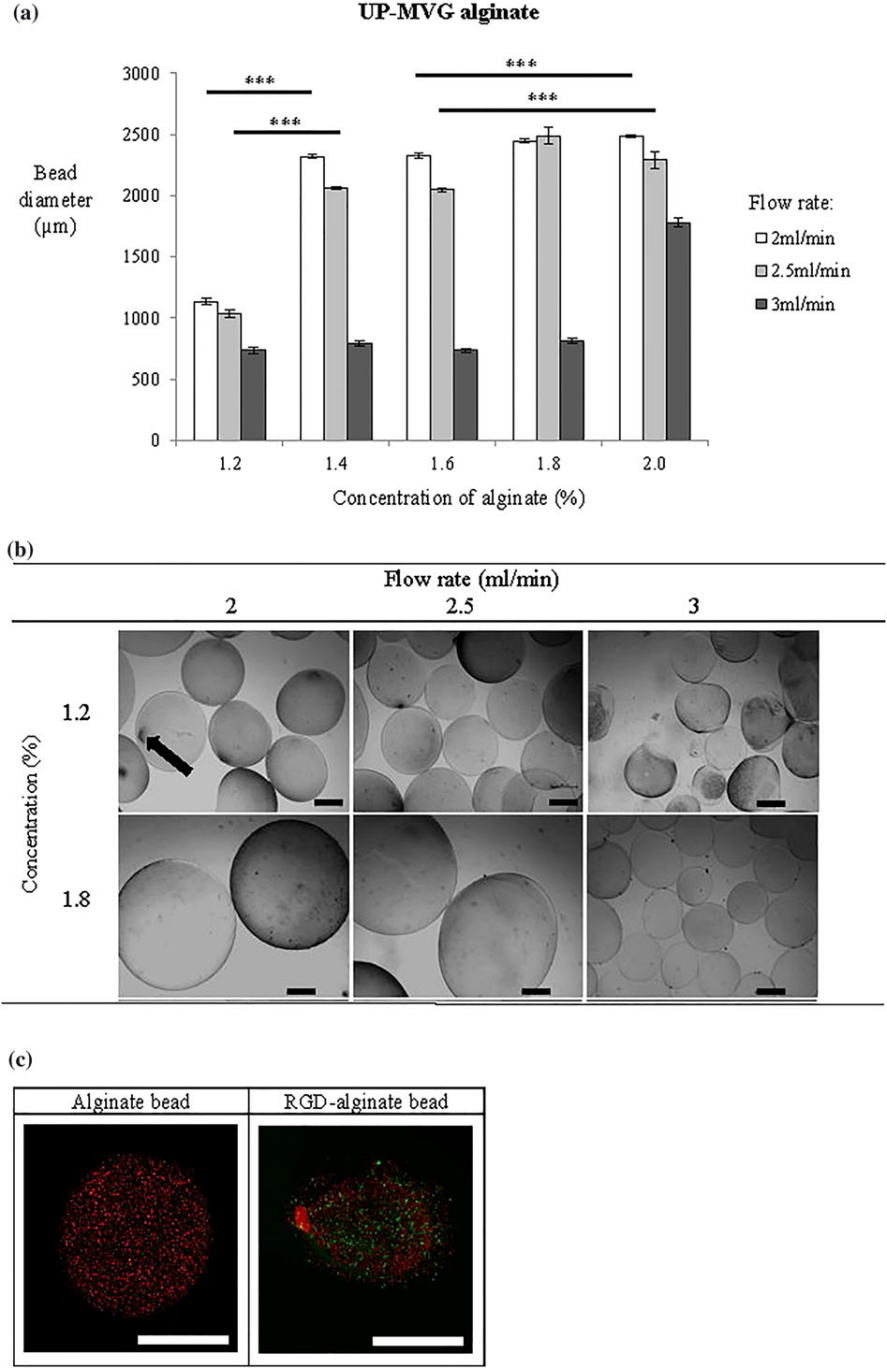
The effect of alginate concentration (%) and flow rate (ml/min) on the diameter of beads derived from high guluronic acid alginate (UP‐MVG, *n* = 50). (a) Graph showing the effect of alginate concentration and solution flow rate though the needle on bead diameter and roundness. The smallest diameter alginate beads with spherical morphology were formed using the highest flow rate (3 ml/min) at a solution concentration of 1.8% (*N* = 3). Significance was tested by two‐way ANOVA *** = *p* < 0.001; error bars denote standard error of the mean (± SEM). (b) Brightfield microscopy images showing the physical morphology of UP‐MVG alginate beads synthesised at different concentrations of alginate and different flow rates. Formation of satellite fraction or tails (arrow) appeared at the lowest concentration of 1.2%. Figure shows representative images depicting the entire population of alginate beads (*n* = 15) (Nikon Eclipse T_*i*_‐E, Japan). (c) Fluorescent microscopy images representative of HUES2 cells cultured in alginate beads after 14 days. HUES2 cells showed higher viability in RGD‐alginate modified beads than alginate beads, and viable cells appeared in very small aggregates. Calcein‐AM stained for live cells (green); propidium iodide, PI stained for dead cells (red). Images were captured using confocal microscopy (Zeiss‐LSM 710) and processed with ZEN light 2011 software. Scale bar: 500 μm [Colour figure can be viewed at wileyonlinelibrary.com]

Alginate beads were optimised to produce suitable bead diameter and morphology for 3D culture of hPSCs. Diameters ranging from 700–2,500 μm were obtained from alginate derived from high guluronic acid or G content (UP‐MVG; Figure 3a). Bead diameter was adjusted by changing alginate concentrations and flow rates. A significant correlation between the two parameters was identified: The higher the flow rate, the smaller the bead diameter. The bead diameter increased constantly and proportionally to concentration at lower flow rates (2 and 2.5 ml/min). The data indicated that the highest flow rate, set at 3 ml/min in the study, played a critical role in reducing the bead diameter in all concentrations. However, the bead morphology and satellite fraction content were modulated by the concentration. The beads were produced with rounder shapes at higher concentrations than at lower concentrations, whereas the formation of satellites (tails) was only found at 1.2% (Figure 3b).

Small beads with diameters of less than 1,000 μm are favourable, decreasing the diffusion distance to the center of the bead and increasing the surface‐to‐volume ratio. Use of alginate derived from a 1.8% concentration solution and at a flow rate of 3 ml/min was found to produce beads with an average diameter 800 μm was chosen as optimal to satisfy the quality requirements of bead integrity, size, spherical morphology, and with no presence of satellite fraction.

### 3.4 Cell viability increased in RGD functionalised alginate

**Figure 4:**
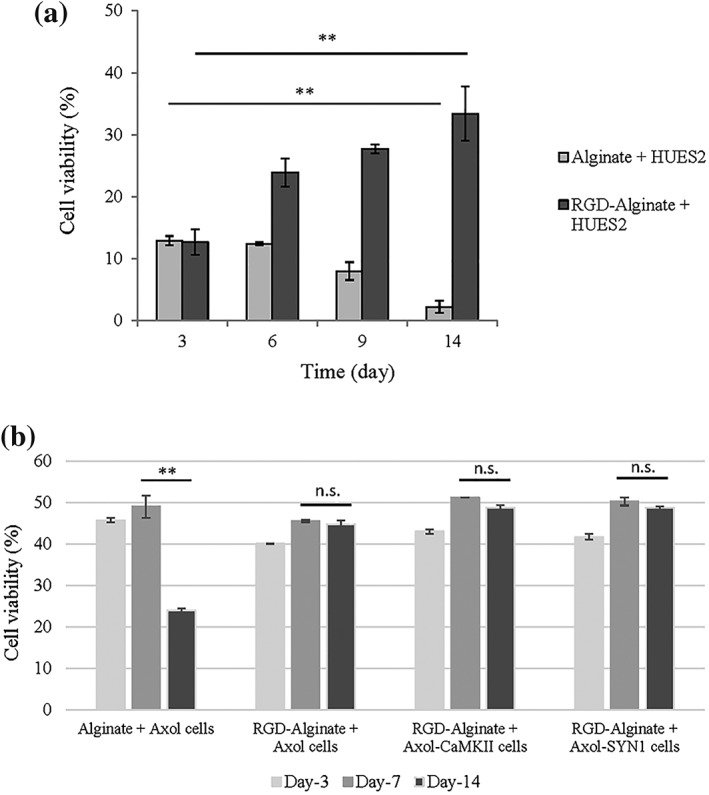
3D culture of hESCs (HUES2) and hiPS‐NPC (Axol cells) in RGD‐alginate showed higher viability than unmodified alginate in 14 days of culture. (a) RGD‐alginate beads increased cell viability of encapsulated HUES2. (b) Axol and Axol‐ChR2 cells demonstrated higher viability in RGD‐alginate than alginate. Live cells were stained with Calcein‐AM (green); dead cells were stained with propidium iodide, PI (red). Images were captured using confocal microscopy (Zeiss‐LSM 710) and processed with ZEN light 2011 software. The percentage of surviving cells was determined by the number of green signals divided by the total number of cells (green and red signals) and analysed using a sectional‐views in ImageJ software. Significance was tested by ANOVA; ** = *p* < 0.01; n.s. denoted a non‐significant value and error bars represent standard deviation (± SD)

Cells were initially encapsulated within alginate at a density of 2 × 10^6^ cells/ml, and the total viable cells were measured by live‐dead cell staining along the time course. The growth of HUES2 is very sensitive to the microenvironment and changes within the cell niche (Gattazzo, Urciuolo, & Bonaldo, 2014). Functionalised alginate hydrogels with RGD in this study appeared to support the viability of HUES2. Cell viability of HUES2 in RGD‐alginate was significantly increased (*p* < 0.01) on Day 14 when compared with unmodified alginate and to the early stage of 3D culture (Day 3; Figure 4a). The results demonstrated that conjugated RGD peptide enhanced HUES2 attachment and cell survival in the alginate beads, in agreement with previous studies also using hPSCs (Hwang, Varghese, Zhang, & Elisseeff, 2006; Lee & Mooney, 2001). The pluripotency of HUES2 was evaluated for OCT‐4, NANOG, ALP, and SSEA‐4 (Positive expressions are reported in Supplementary Figure 2A and 2B). The HUES2 cells exhibited expression of βIII‐tubulin at Day 14 and Day 21 after neural differentiation (Supplementary Figure 2C).

Axol cells showed higher cell viability than HUES2 in the RGD‐alginate system. Cell viability increased and was maintained at between 45 and 50% at Day 7 and Day 14 in the culture (Figure 4b). The viability of Axol cells encapsulated in alginate without RGD was similar to that observed for HUES2 cells, dropping significantly by Day 14 (*p* < 0.01).

### 3.5 ChR2‐eYFP expression remained when culture in 3D RGD‐alginate

**Figure 5:**
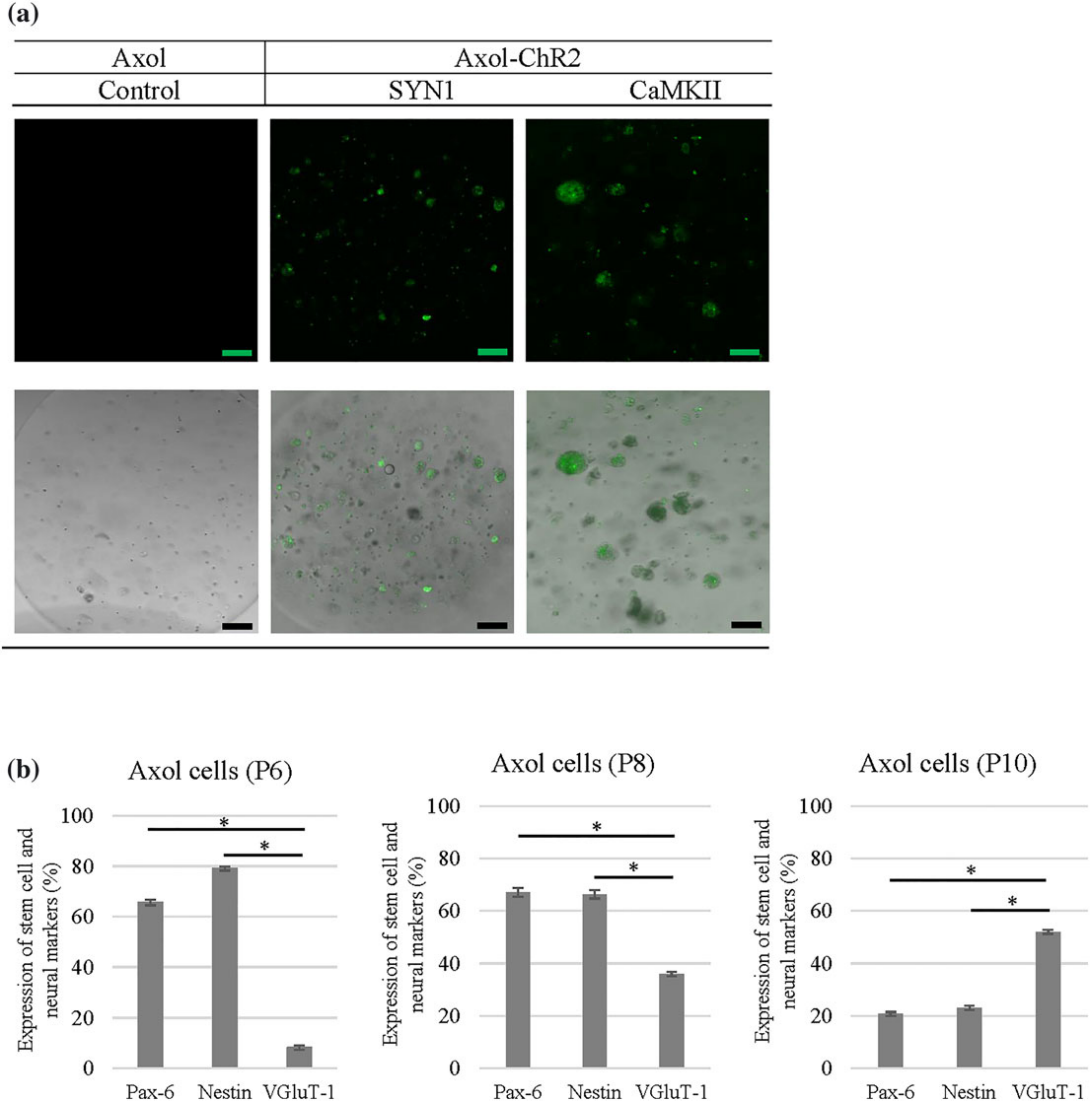
ChR2‐eYFP positive expression of Axol cells differentiated from hiPSC‐NPC was retained in the 3D culture system. (a) Top: Microscopy images showing fluorescent tagged ChR2 expressing cells (green) inside single alginate beads. Optogenetically modified Axol cells cultured in 2D were enzymatically detached and encapsulated within RGD‐alginate beads. The constructs were examined using a confocal microscope (Zeiss‐LSM 710) to investigate the expression of ChR2‐eYFP, cell morphology and distribution within the 3D culture system. Non‐transduced Axol cells were used as a control. (a) Bottom: Merged images showing fluorescent images combined with brightfield phase‐contrast images; scale bars: 100 μm. (b) Graphs showing how the maturation of neurons increased during passaging (P6–P10) in neural differentiation medium. The heterogeneous Axol cells were harvested from different passages to determine cell phenotype spread within their cell populations using flow cytometry (*N* = 3). The cells were stained with progenitor stem cell markers (Pax‐6 and Nestin) and neural marker (vGluT1) to assess neuronal maturation over passaging and culture. Unstained cells were used as a control and to set gate regions. ANOVA was used to assess significance. A *p‐value below 0.05 was considered statistically significant. Error bars indicate standard deviation (± *SD*) [Colour figure can be viewed at wileyonlinelibrary.com]

Optogenetically modified neurons (Axol‐ChR2 cells) were further investigated for their potential to establish a 3D culture network culture model by encapsulating these cells into RGD‐alginate beads. Confocal microscopy revealed that expression of eYFP‐ChR2 by transduced Axol cells was not affected by the culture environment, or by bead diameter (Figure 5a). The Axol‐ChR2 cells were found clustered in spherical aggregates within the RGD‐alginate beads, however no outgrowth of neurites was observed after 24 hrs of encapsulation. The cells were characterised using progenitor and neural markers at different passages. Upon neural differentiation, Axol cells at Passage 10 showed higher expression of mature neurons (VGluT‐1) with approximately 50% (*p* < 0.5) when compared with Pax‐6 and Nestin (Figure 5b).

### 3.6 Neural network‐forming capability on 2D and 3D culture

**Figure 6:**
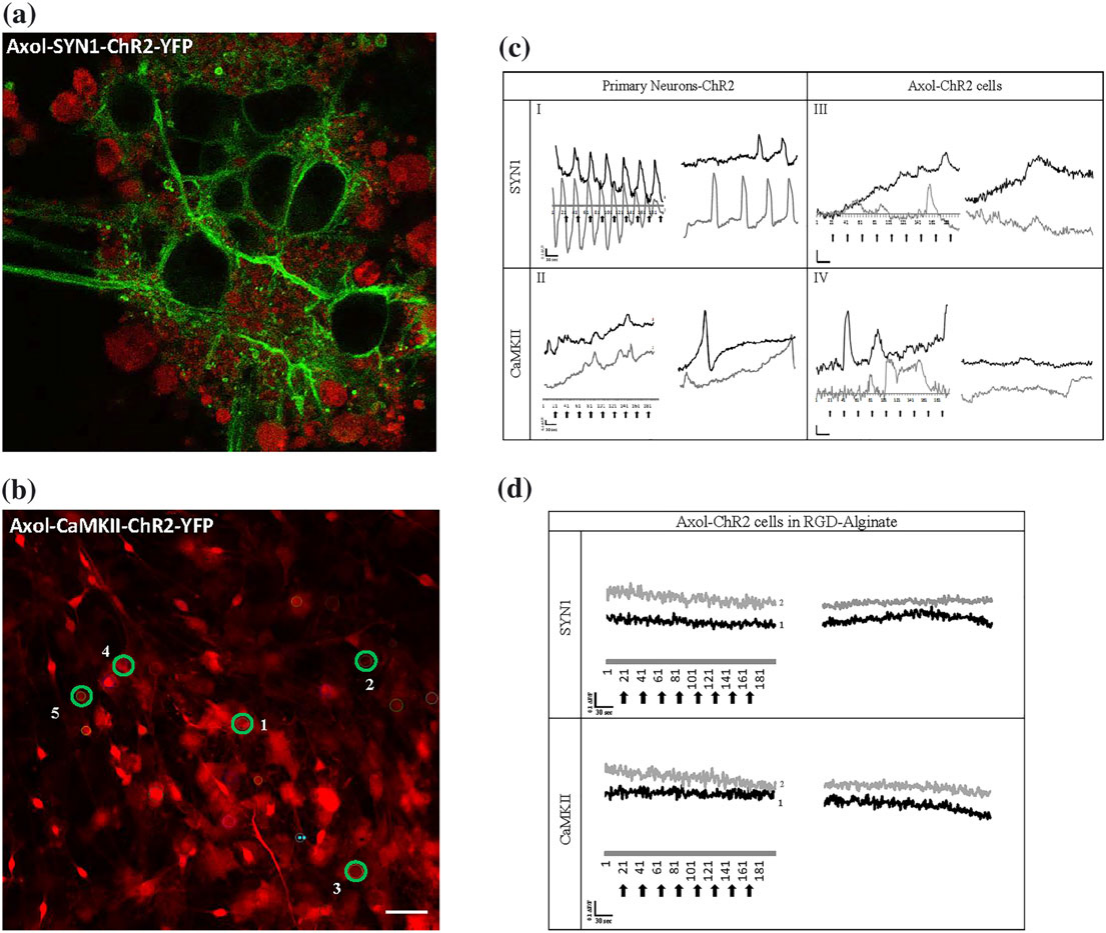
Response of Axol cells expressing ChR2‐eYFP driven by both SYN1 and CaMKII promoters to light stimulation. (a) High magnification image of Axol cells transduced to express ChR2‐eYFP localised in the membrane (green) and loaded with CAL‐590 calcium indicator (red). Samples were imaged using confocal microscopy at an excitation wavelength of 540/565 nm (Zeiss‐LSM 710). Magnification: 40x. (b) Lower magnification image showing CAL‐590 calcium dye stained Axol cells cultured in 2D, with five regions of interest (ROI) marked (green circles). Scale bar: 50 μm. (c) Calcium flux traces of primary neurons and Axol cells, and (d) calcium flux traces of Axol cells in RGD‐alginate expressing ChR2‐eYFP loaded with CAL‐590 (calcium dye showing traces from two regions of interest for (c) and (d), black line: ROI‐1 and grey line: ROI‐2. The cells were optically stimulated with a laser at 488 nm every 30 sec (200 frames), and the fluorescence intensity was normalised to the level of baseline fluorescence measured before the onset of the calcium signal (∆F/F). Scale = 0.1 ΔF/F (y‐axis); 30 sec (x‐axis); black arrows: light stimulation; scale bar: 50 μm [Colour figure can be viewed at wileyonlinelibrary.com]

The neural network‐forming capability of optically excitable iPSC‐derived neurons (Axol‐ChR2 cells) in 2D cultures and 3D encapsulated in the RGD‐alginate hydrogel was evaluated using calcium imaging. The action potentials (AP) of the cells were detected with Cal‐590 dye (red) whereas the green fluorescence signal indicated the location of ChR2‐eYFP expression at the membrane and within the neurites of the cells (Figure 6a). In 2D culture, optogenetically modified primary neurons exhibited active and repetitive firing upon light stimulation under the control of SYN1 and CaMKII promoters (Figure 6b,c). Similar results were obtained in Axol‐ChR2 cells, however, higher neuronal activity was observed in cells with the expression of ChR2 driven by the CaMKII promoter. These experiments indicate successful neuronal specific cell targeting using optogenetics.

**Figure 7:**
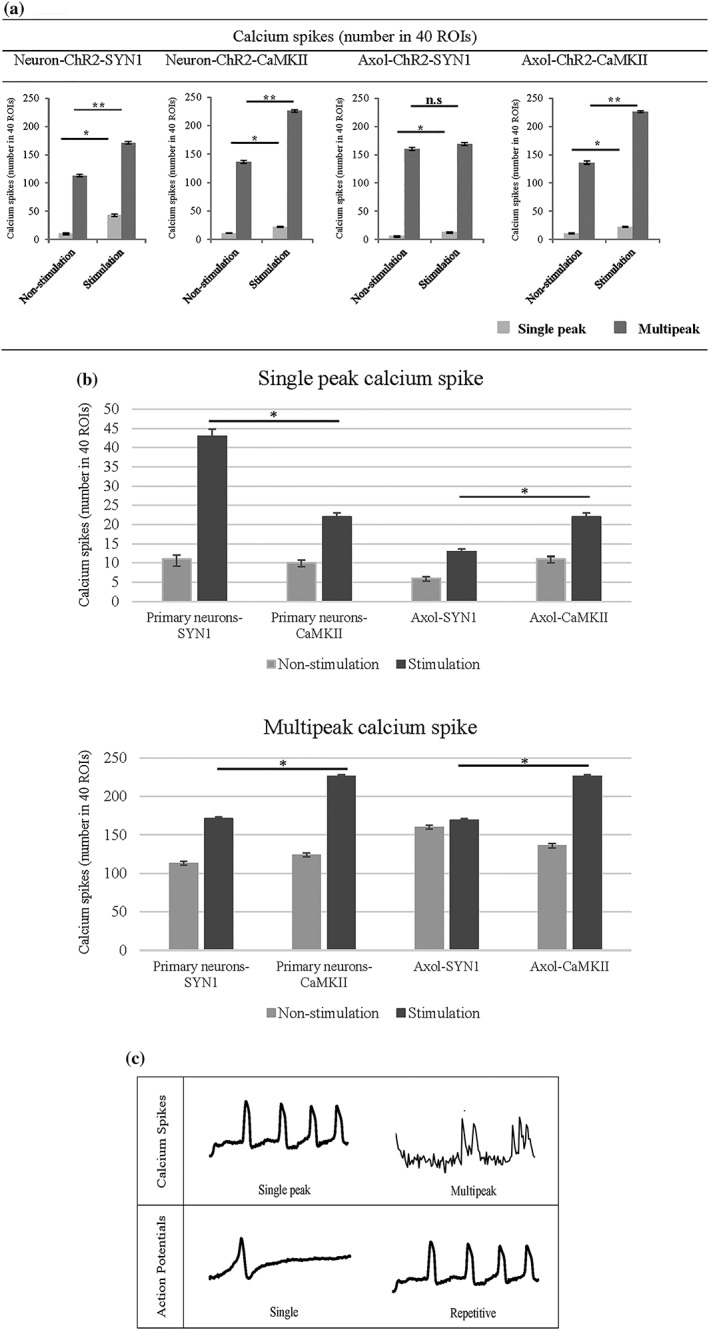
An increase in both the number of single peak and multipeak calcium spikes was observed in optogenetically modified primary neurons and Axol cells upon optical stimulation. Although the CaMKII promoter was found to drive less overall ChR2 expression than the SYN1 promoter, CaMKII driven expression was targeted at mature neurons that responded to light stimuli. (a) Calcium waves were analysed from the ROIs of nonstimulated and stimulated cells derived from transduced primary neurons (Neuron‐ChR2‐SYN1 and Neuron‐ChR2‐CaMKII) and Axol cells (Axol‐ChR2‐SYN1 and Axol‐ChR2‐CaMKII; ROIs = 40, *n* = 3). A higher number of burst waves and multipeak calcium spikes was found to be driven by cells expressing ChR2 containing CaMKII promoter upon stimulation, indicating the presence of mature neurons. Calcium spikes (single peak and multipeak) were identified from the selected 40 ROIs. Significance was assessed by two‐way ANOVA * = *p* < 0.05; *ns* denoted a non‐significant value and error bars represent standard deviation (± *SD*). (b) Comparison of single peak and multipeak calcium spikes, respectively in primary neurons and Axol cells (analysed from the same samples and ROIs used in (a)). (c) Examples of different trace categories, distinguished as (1) single peak and multipeak, calcium spikes and (2) single or repetitive calcium transient action potentials. The traces represent typical examples of calcium imaging time series over 5 mins from different ROIs

Further measurement demonstrated that the number of calcium spikes, consisting of both single and multipeak spikes, increased significantly upon stimulation in both primary neurons expressing ChR2 (driven by SYN1 and CaMKII promoter) and Axol‐ChR2 cells (driven by CaMKII with mostly single spikes generated by cells expressing ChR2 driven by the SYN1 promoter; Figure 7a). Upon light stimulation, primary neurons with ChR2 expression driven by the SYN1 promoter showed a higher number of single peak and multipeak calcium spikes in comparison to primary neurons with ChR2 expression driven by the CaMKII promoter. In contrast, higher calcium flux activity was driven by the CaMKII promoter in Axol cells (Figure 7b). A single peak represents a complete AP. The type of calcium peak and AP is presented in Figure 7c whereas the classification of calcium event is described in Supplementary Figure 3.

**Figure 8:**
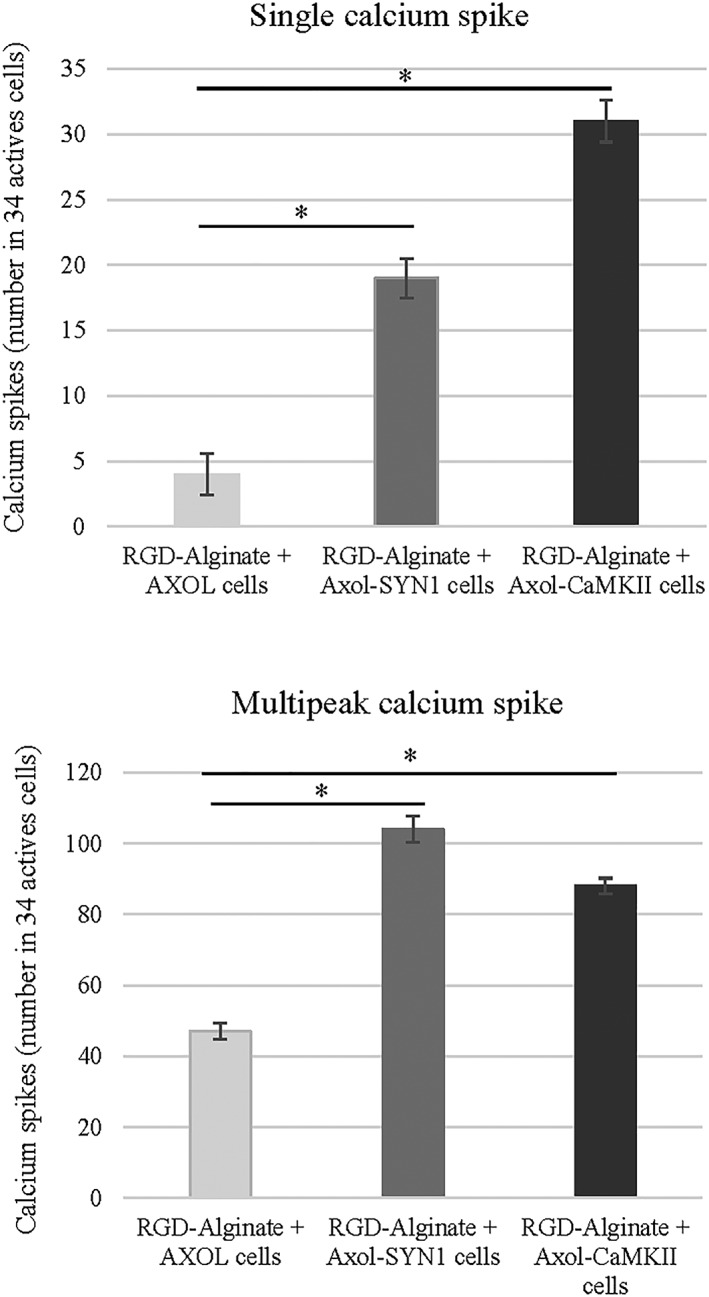
Upon light stimulation, an increased number of calcium spikes (single peak and multipeak) was observed in Axol‐ChR2 cells driven by SYN1 and CaMKII promoter, indicating functional activity achieved in a 3D neural model using RGD‐alginate. The optogenetically modified cells (Axol‐ChR2‐SYN1 and Axol‐ChR2‐CaMKII) and unmodified Axol cells were encapsulated in the alginate bead system (RGD‐ALG), respectively. The cell constructs were stained with calcium dye and imaged using confocal microscopy (Zeiss‐LSM 710). Total of 34 active cell aggregates were selected from the ROIs (*N* = 3) and stimulated with light before further analysed for the number of calcium spikes. Significance was tested by two‐way ANOVA * = *p* < 0.05; error bars represent standard deviation (± *SD*)

Calcium imaging recordings revealed that neural activities of light‐stimulated Axol‐ChR2 cells in the RGD‐alginate hydrogel appeared in mixed and burst calcium waves, whereas non‐stimulated cells exhibited slow undefined waves (Figure 6d). Upon stimulation, the number of calcium spikes (single peak and multipeak) increased significantly, driven by the SYN1 and CaMKII promoters (Figure 8). A higher number of single peak spikes was recorded in encapsualted Axol‐ChR2 cells driven by the CaMKII promoter, thought to indicate the presence of a greater number of functionally mature neurons in the culture.

## 4 DISCUSSION

In this study, we demonstrated that the human iPSCs derived neural progenitor cells successfully differentiated into neurons that expressed ChR2 driven by the neuronal specific SYN1 and CaMKII promoters. The expression of ChR2 under the control of the SYN1 and CAMKIII promoters, maturation, and electrical activity of the optogenetically engineered neurons were evaluated in both 2D cultures and 3D hydrogel cultures.

The delivery of ChR2‐eYFP into human iPSCs derived neurons was mediated by lentiviruses. Transduction at MOI‐2 and MOI‐1 followed by re‐infection did not induce significant cell death but achieved high expression of ChR2‐eYFP. Both cytosolic eYFP and membrane‐bound ChR2 were localised throughout the entire cell (somata and neurites). Similar results have been demonstrated by Uzel and colleagues in the optogenetic targeting of ESC and the optical excitability of ChR‐H134R‐ESC‐derived motor neurons (Uzel et al., 2016). Furthermore, Rapti and colleagues have compared the major viral vectors of adeno‐associated viruses, adenoviruses, and lentiviruses using various undifferentiated cells (hPSCs: hES2, H9, hiPS31.3, hiPS24.1) and differentiated cells (cardiomyocyte derivatives). Their findings agreed that lentiviral vectors transduced all cell types with moderate efficiency (Rapti et al., 2015). Other research groups have reported that ChR2‐ESC‐derived neurons displayed strong ChR2‐expression, mature neuronal morphology, and positive expression of vGlut2 marker (Stroh et al., 2011), and this is in agreement with our findings from the use of lentivirus transduction on ChR2‐iPSC‐derived neurons (Axol‐13 cell line). Other studies have also reported the robust expression of SYN1 promoter in various types of neuronal cells including hPSC‐derived neurons (Steinbeck et al., 2015).

Following transduction, human iPSC derived neural progenitor cells were differentiated to distinct neuronal phenotypes with positive expression of neuron‐specific tubulin (TuJ1) and astrocytes markers (S100B/GFAP). Mature glutamatergic and GABAergic neuronal subtypes, were observed, indicating the presence of excitatory and inhibitory neurons. Although optogenetic approaches have recently been used for in vivo and in vitro study in neuroscience (Steinbeck et al., 2015), it is novel to apply this strategy to generate an in vitro 3D neural culture model. Furthermore, the 3D culture system developed using modified alginate hydrogels (alginate functionalised with RGD and ROCKi showed potential in supporting cell survival and allowing neural networks to be light‐stimulated in 3D culture.

Prior to culture with cells, the physical properties of alginate hydrogel (bead size, sphericity and consistency of formation) were characterised. Results revealed that the physical properties of the hydrogel correlate to chemical composition, and specifically to the proportion of guluronic to mannuronic acid residues in alginate. Alginate consisting of a higher guluronic acid and purity (UP‐MVG) forms stiffer gels and rounder beads, and this enables the physical properties of alginate to be maintained for a longer period of culture. In line published reports, it was found that microspheres produced from highly purified alginate has less morphological imperfections, resulting in the production of more spherical beads with smaller diameters (Kendall, Darrabie, El‐Shewy, & Opara, 2004). Although increasing the concentration of alginate correlated directly with an increase in bead size, flow rate was found to exert a significant influence on bead diameter. The bead diameter was optimised for cell encapsulation and culture, to enable rapid nutrient and gas diffusion through the alginate. Using the optimised parameters, the parameters alginate bead diameter (800 μm) falls within the range of 600–1,000 μm, where glucose, ammonia, and vitamin B have been found to rapidly diffuse across the outer layer of the bead (Gautier et al., 2011).

Other bead production parameters such as needle size also contributes to the shape and size of alginate beads. A larger diameter needle (25G, 5/8″) may lead to bead deformation (tailing), which is a very common phenomenon found with the use of viscous alginate solutions (Fundueanu, Nastruzzi, Carpov, Desbrieres, & Rinaudo, 1999). When a smaller diameter needle (30G, 1/2″) was used for extrusion of the beads, the beads with the smallest diameter could be formed with no tailing in combination with use of alginate formulations of high concentration (1.8%) with high extrusion flow rate (3 ml/min).

After encapsulation, HUES2 cells in RGD‐modified alginate demonstrated higher viability than those encapsulated in unmodified alginate, as shown by live/dead analyses. This is thought to be because the RGD sequence acts as a site to bind cell‐membrane integrin receptors, resulting in increased cell attachment and viability. It has previously been shown that bioadhesive sequences, such as RGD and IKVAV, can increase cell adhesion and growth through the integrin‐mediated pathway (Li et al., 2014; Villard et al., 2006). One cause of cell death in the functionalised hydrogels may be apoptosis. Our findings suggest that whilst alginate hydrogel may support the culture of other cell types, alginate alone is unable to provide an optimal microenvironment for the growth of hPSCs (HUES2). In this study, Axol cells survived better than HUES2 in RGD‐alginate. Alginate provides a promising option for 3D cell cultures with cell viability at 40–50%. Alginate is a commonly chosen 3D culture substrate, having favorable characteristics and properties including its ability to make hydrogels at physiological conditions, transparency for microscopic evaluation, penetration of light for optical stimulation, gentle dissolution for cell retrieval, pore networks that allows diffusion of nutrient and waste as well as its nonanimal origin (Andersen, Auk‐Emblem, & Dornish, 2015).

Interestingly, encapsulated Axol‐ChR2 cells in RGD‐alginate beads remained in aggregates with minimal neurite extension. Similar results have been observed elsewhere, where the incorporation of the IKVAV peptide sequence did not lead to an increase in neurite length in silk fibroid hydrogel after 1 week of differentiation (Sun et al., 2017). This could be due to structural limitations of the hydrogel, and pore size should be optimised for the migration and neurite outgrowth of cells in the future. This study suggests a potential new application of RGD‐alginate hydrogels in brain tissue engineering. To further improve alginate hydrogels to support neurite extension it is suggested that pore size could be optimised. To improve cell attachment, a further approach could be to immoblise different kinds of peptides in the alginate hydrogel to increase cell adhesion and neurite outgrowth.

When the cells were transferred from 2D to 3D culture, the expression of ChR2‐eYFP was found to be stable in the RGD‐alginate hydrogels, suggesting an initial success towards the development of a functional 3D neuronal culture model that may respond to light stimulation. Results show that the cells remained alive inside the RGD‐alginate hydrogel, and their calcium activities were detectable. Prior to encapsulation inside the hydrogel, confocal imaging revealed that the neurons were able to extend neurites that formed extensive networks in 2D culture, and that the cells differentiated into neurons and achieved maturation. Their neural function and response to light stimulation was confirmed. Results were also similar to previous studies reported on murine ESC‐ and hESC‐derived neurons, which display basic neurophysiological activity in culture, such as AP firing and synaptic currents (Heikkila et al., 2009; Johnson, Weick, Pearce, & Zhang, 2007).

Upon light stimulation, a normal repetitive pattern of neural activity was shown in primary mouse neurons (E14.5) indicating that membrane depolarisation could be triggered by light with ChR2 expression driven by the chosen neuron specific promoters. Expression of ChR2 in both inhibitory neurons and excitatory neurons was successfully driven by SYN1 and CaMKII promoters, respectively. When compared with spontaneous calcium activity of non‐stimulated primary mouse neurons, non‐stimulated Axol‐ChR2 cells have generated a lower number of repetitive APs and mixed waves. These results imply that neural function of Axol cells may require longer time to mature after being transduced (optogenetically modified) and passaged in the culture. These cells were differentiated from hiPSCs, which also possibly contained early formed neurons with less AP firing than primary mouse neurons, higher multipeak calcium spikes than single peaks as well as slow or mixed calcium waves. The immature nature of human neural stem cell cultures has been observed and reported by other laboratories (Patani et al., 2012). The Axol‐ChR2 cells in RGD‐alginate showed excitability upon light stimulation, with mixed and burst calcium waves recorded. Cell encapsulation is likely to affect calcium activity and light penetration, further investigation is required. In Axol‐ChR2 cells, ChR2 expression driven by the CaMKII promoter (associated to glutamatergic neurons) resulted in higher levels of calcium activity and calcium spikes even though CaMKII was a weaker promoter than SYN1. As a pan‐neuronal promoter, SYN1 has been commonly used in many studies. For example, Weick and colleagues have utilised the SYN1 promoter (Kugler, Kilic, & Bahr, 2003) to drive ChR2 expression in hESC‐derived neurons with a variety of neurotransmitter phenotypes. Light stimulation of these cells expressing ChR2 could reliably drive high AP frequencies (5–30 Hz) depending on cell maturity. They reported that ChR2 mediated light stimulation induced post‐synaptic currents in glutamatergic and GABAergic neurons both in vitro and in vivo (within transplanted tissue) for at least 6 months (Weick et al., 2010). Despite lentiviral transduction, successful ChR2 expression within Axol cells driven by CaMKII in this study was possibly due in part to increased neural maturation achieved through use of higher passages and longer period of culture.

The aim to create in vitro models of neural networks that closely resemble those found in vivo has been a challenge. Several groups have worked on efficient neural differentiation of hiPSCs but mostly this has taken place in 2D culture systems (Shi, Kirwan, Smith, Robinson, & Livesey, 2012). Only a few studies described the 3D hydrogel culture of dissociated neural stem/progenitor cells derived from hPSCs (Alessandri et al., 2016; Sun et al., 2017). We report a novel strategy to combine the use of RGD‐alginate hydrogel and optogenetic approach to generate a 3D in vitro model of human neural networks. Our findings demonstrated that hiPSC‐derived neurons (Axol‐ChR2) exhibit the expression of ChR2‐eYFP and are responsive to light stimulation (optically excitable) when encapsulated in the RGD‐alginate beads. Moreover, a high number of calcium spikes (both single and multipeak) was observed, driven by the CaMKII and SYN1 promoter upon light stimulation, indicating the presence of functional mature neurons. The expression triggered by CaMKII facilitates identification of glutamatergic excitatory neurons, whereas SYN1 has efficiently labelled GABAergic inhibitory neurons. The transparent 3D RGD‐alginate systems not only support survival of Axol‐ChR2 cells but also permit penetration of light for ChR2‐eYFP activation, as well as allow recording of neurophysiological activity through the visualisation of calcium transients concentration changes using calcium imaging. Further electrophysiology analysis of functional connectivity and electrical properties of the 3D neural networks in RGD‐alginate beads could be investigated using multielectrode array recording in the future (Heikkila et al., 2009).

## 5 CONCLUSIONS

In the current study, we show that hiPSC‐derived neuronal cells are successfully generated and optogenetically engineered. The expression of ChR2 is efficiently driven by neuron‐specific promoters CaMKII and SYN1, indicating the potential to target within the excitatory and inhibitory neural populations. The ChR2‐positive neurons are not only viable but also detectable inside a transparent RGD‐alginate hydrogel using calcium imaging. In conclusion, the cell‐biomaterial construct created in this study combined with the optogenetic approach leads to the establishment of a functional 3D neural network model, which can be used for future drug screening purposes, non‐invasive repetitive functionality analysis and neuromodulation, as well as providing new information for neural tissue engineering and stem cell research.

## CONFLICT OF INTEREST

The authors declare no conflicts of interest.

## DATA SHARING

Plasmids and protocols used in this work are available on request. The research materials supporting the findings of this study are available within the article and its supplementary information files.

## Supporting information


**Figure S1.** Comparison of the promoters (synapsin‐1, calcium‐calmodulin kinase II, and elongation factor‐1 alpha) in channelrhodopsin‐2 (ChR2) expression (%) at different viral transduction condition (a) multiple of infection‐1 and (b) multiple of infection‐2. The quantification of ChR2 expression was performed using flow cytometry (*N* = 3). Significance was tested by ANOVA; * = *p* < 0.05; error bars denote standard error of deviation (± SD).
**Figure S2.** The human embryonic stem cells (HUES2) expressed pluripotent markers at the time of encapsulation. (a) The cells were fixed in 4% paraformaldehyde in PBS for 30 min at room temperature (RT), washed with PBS, and then stained with OCT‐4, NANOG, ALP, and SSEA‐4 (R&D system, Minnesota, United States), respectively for 60 min at RT. Cells were resuspended in blocking donkey serum, permeabilised and incubated with intracellular markers at 4°C in the dark for 30 min. Samples were washed twice carefully in the cold room before being stained with secondary antibody (Fluorescein isothiocyanate (FITC) ‐conjugated goat anti‐mouse, 1:20 and FITC‐conjugated donkey anti‐goat, 1:500, R&D system). The nuclei were counterstained with DAPI (blue), and the cells were imaged using a fluorescence microscope (Nikon Eclipse T_*i*_‐E, Japan). Scale bar: 100 μm. (b) For flow cytometry experiments, cells were incubated with ALP and SSEA‐4 unconjugated antibody (R&D systems) to mark the expression of cell surface antigens. For intracellular staining with OCT‐4 ‐ PerCP‐Cy5.5 and NANOG – PerCP‐Cy5.5 (BD Bioscience), the cells were fixed with 4% paraformaldehyde in PBS for 10 min, washed twice with PBS, and permeabilised with 0.1% saponin in PBS prior to incubation with antibodies. All antibody incubations were performed according to the manufacturer's instructions. Isotype controls were included for each antibody staining, and the emission wavelength of 488 nm was used. Cell Quest Pro software was used for both data acquisition and analysis to produce histogram plots and median peak values. As a control for nonspecific binding for each conjugated antibody, we used the same IgG subclass with the same fluorochrome conjugation and for nonconjugated antibody—the same IgG subclass conjugated to fluorochrome. A total of 10,000 events were acquired for each analysis. (c) The HUES2 cells expressed neural marker β‐III Tubulin at Day 14 and Day 21 of neural differentiation. The cells were imaged at excitation wavelength of 568 nm using a fluorescence microscope (Nikon Eclipse T_*i*_‐E, Japan).
**Figure S3.** Classification of calcium events obtained from calcium imaging. Traces represent typical examples of calcium imaging time series over 5 min from different region of interests, which were classified based on the calcium waves: (a) inactive, (b) slow undefined, (c) slow rise, (d) burst, and (e) mixed.
