# Supplementary material for: Optogenetic control of iPS cell‐derived neurons in 2D and 3D culture systems using channelrhodopsin‐2 expression driven by the synapsin‐1 and calcium‐calmodulin kinase II promoters
Source: J Tissue Eng Regen Med. 2019 Jan 30;13(3):369–84. doi: 10.1002/term.2786 (PMC6492196; doi:10.1002/term.2786)
Supplement: Supplementary file 1 — Figure S1. Comparison of the promoters (synapsin‐1, calcium‐calmodulin kinase II, and elongation factor‐1 alpha) in channelrhodopsin‐2 (ChR2) expression (%) at different viral transduction condition (a) multiple of infection‐1 and (b) multiple of infection‐2. The quantification of ChR2 expression was performed using flow cytometry (N = 3). Significance was tested by ANOVA; * = p < 0.05; error bars denote standard error of deviation (± SD). Figure S2. The human embryonic stem cells (HUES2) expressed pluripotent markers at the time of encapsulation. (a) The cells were fixed in 4% paraformaldehyde in PBS for 30 min at room temperature (RT), washed with PBS, and then stained with OCT‐4, NANOG, ALP, and SSEA‐4 (R&D system, Minnesota, United States), respectively for 60 min at RT. Cells were resuspended in blocking donkey serum, permeabilised and incubated with intracellular markers at 4°C in the dark for 30 min. Samples were washed twice carefully in the cold room before being stained with secondary antibody (Fluorescein isothiocyanate (FITC) ‐conjugated goat anti‐mouse, 1:20 and FITC‐conjugated donkey anti‐goat, 1:500, R&D system). The nuclei were counterstained with DAPI (blue), and the cells were imaged using a fluorescence microscope (Nikon Eclipse Ti‐E, Japan). Scale bar: 100 μm. (b) For flow cytometry experiments, cells were incubated with ALP and SSEA‐4 unconjugated antibody (R&D systems) to mark the expression of cell surface antigens. For intracellular staining with OCT‐4 ‐ PerCP‐Cy5.5 and NANOG – PerCP‐Cy5.5 (BD Bioscience), the cells were fixed with 4% paraformaldehyde in PBS for 10 min, washed twice with PBS, and permeabilised with 0.1% saponin in PBS prior to incubation with antibodies. All antibody incubations were performed according to the manufacturer's instructions. Isotype controls were included for each antibody staining, and the emission wavelength of 488 nm was used. Cell Quest Pro software was used for both data acquisition and analysis to produce [file TERM-13-369-s001.docx]

A

Time (Day)

ChR2 expression (%)

B

Time (Day)

ChR2 expression (%)

**Figure S1: Comparison of the promoters (SYN1, CaMKII and EF1a) in ChR2 expression (%) at different viral transduction condition (A) MOI-1 and (B) MOI-2.** The quantification of ChR2 expression was performed using flow cytometry (N = 3). Significance was tested by ANOVA; * = p < 0.05; error bars denote standard error of deviation (± SD).

A

| **OCT-4**  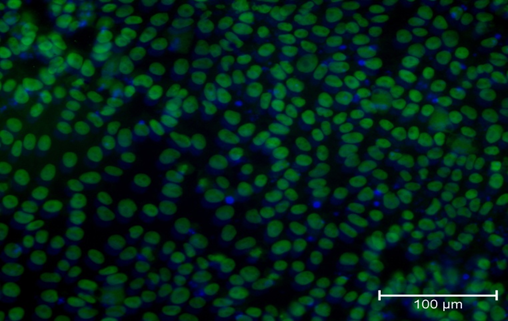 | **Nanog**  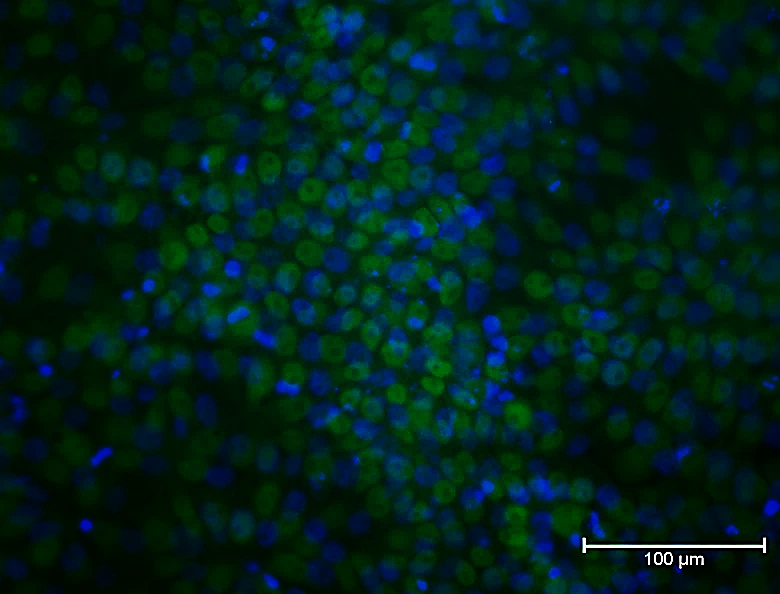 |
| --- | --- |
| **ALP**  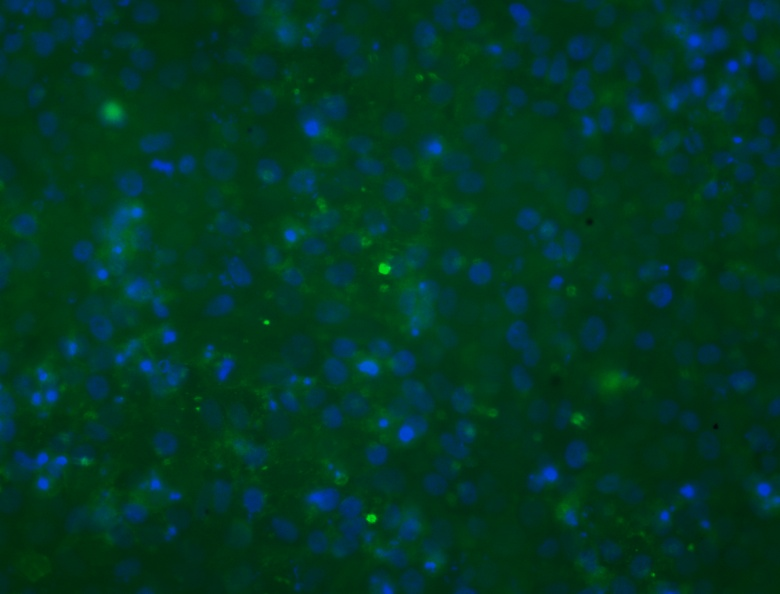  100 µm | **SSEA-4**  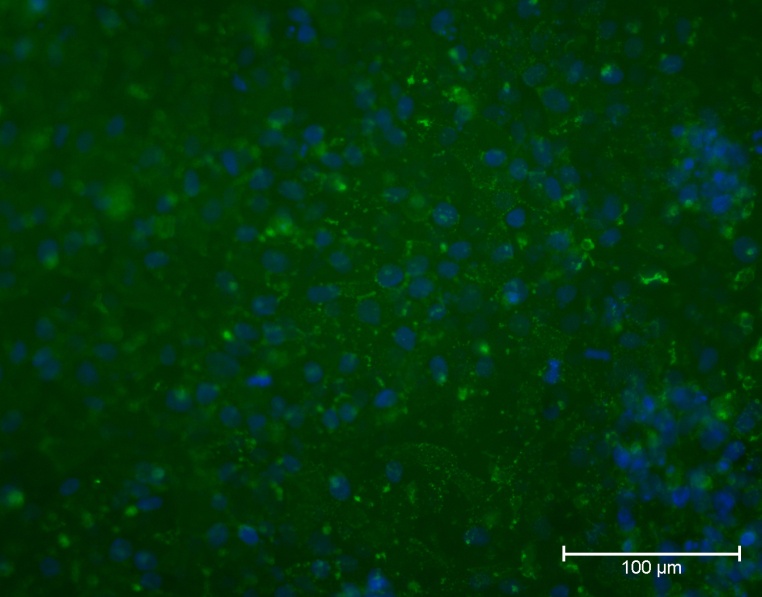 |

B

|   OCT-4  97.2% |   NANOG  81% |
| --- | --- |
|   ALP 95.4% |   SSEA-4 89.6% |

| Pluripotency Markers | Expression |
| --- | --- |
| OCT-4 | 96.3 % (±1.2) |
| NANOG | 83.2 % (±3.0) |
| ALP | 94.8 % (±1.6) |
| SSEA-4 | 90.3 % (±1.8) |

C

**Day-21**

**Day-14**

**Day-21**


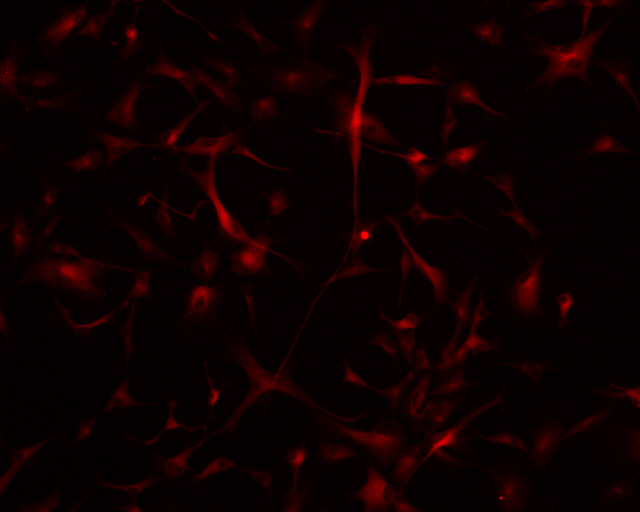

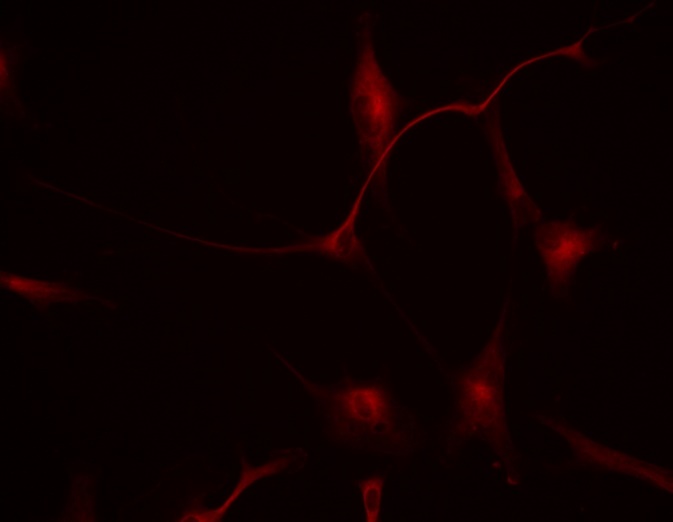


**Figure S2: The human embryonic stem cells (HUES2) expressed pluripotent markers at the time of encapsulation.** (A) The cells were fixed in 4% paraformaldehyde in PBS for 30 min at RT, washed with PBS and then stained with OCT-4, NANOG, ALP and SSEA-4 (R&D system, Minnesota, USA), respectively for 60 min at RT. Cells were resuspended in blocking donkey serum, and permeabilised prior incubated for intracellular markers at 4ºC in the dark for 30 min. Samples were washed twice carefully in the cold room before stained with secondary antibody (FITC - conjugated goat anti-mouse, 1:20 and FITC - conjugated donkey anti-goat, 1:500, R&D system). The nuclei were counterstained with DAPI (blue), and the cells were imaged using a fluorescence microscope (Nikon Eclipse T*_i_*-E, Japan). Scale bar: 100 µm. (B) For flow cytometry experiments, cells were incubated with ALP and SSEA-4 unconjugated antibody (R&D Systems) for the expression level of cell surface antigens. For intracellular staining with OCT-4 - PerCP-Cy5.5 and NANOG – PerCP-Cy5.5 (BD Bioscience), the cells were fixed with 4 % paraformaldehyde in PBS for 10 min, washed twice with PBS and permeabilised with 0.1 % saponin in PBS prior to incubation with antibodies. All antibody incubations were performed according to the manufacturer's instructions. Isotype controls were included for each antibody staining and the emission wavelength of 488 nm was used. Cell Quest Pro software was used for both data acquisition and analysis to produce histogram plots and median peak values. As a control for nonspecific binding for each conjugated antibody we used the same IgG subclass with the same fluorochrome conjugation and for non-conjugated antibody — the same IgG subclass conjugated to fluorochrome. A total of 10,000 events were acquired for each analysis. (C) The HUES2 cells expressed neural marker β-III Tubulin at day-14 and day-21 of neural differentiation. The cells were imaged at excitation wavelength of 568 nm using a fluorescence microscope (Nikon Eclipse T*_i_*-E, Japan).

| Inactive  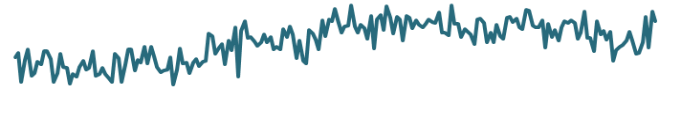  Slow undefined  Slow rise  Burst  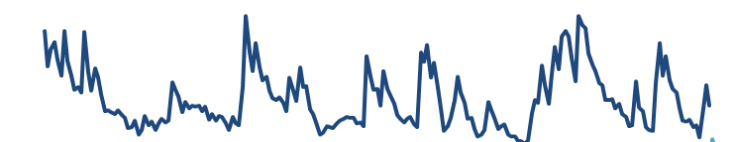  Mixed |
| --- |

**Figure S3**: **Classification of calcium events obtained from calcium imaging**

Traces represent typical examples of calcium imaging time series over 5 min from different ROIs which were classified based on the calcium waves: (i) inactive, (ii) slow undefined, (iii) slow rise, (iv) burst and (v) mixed.
